# A259 METABOLIC DYSFUNCTION-ASSOCIATED STEATOTIC LIVER DISEASE IS ASSOCIATED WITH MULTI-ORGAN COMORBIDITIES AND FIBROSIS PROGRESSION IN PATIENTS WITH INFLAMMATORY BOWEL DISEASE (IBD)

**DOI:** 10.1093/jcag/gwad061.259

**Published:** 2024-02-14

**Authors:** D Kablawi, S Sasson, F Aljohani, C S Palumbo, A Bitton, W Afif, P L Lakatos, G Wild, T Bessissow, G Sebastiani

**Affiliations:** McGill University Health Centre, Montreal, QC, Canada; The University of British Columbia, Vancouver, BC, Canada; McGill University Health Centre, Montreal, QC, Canada; McGill University Health Centre, Montreal, QC, Canada; McGill University Health Centre, Montreal, QC, Canada; McGill University Health Centre, Montreal, QC, Canada; McGill University Health Centre, Montreal, QC, Canada; McGill University Health Centre, Montreal, QC, Canada; McGill University Health Centre, Montreal, QC, Canada; McGill University Health Centre, Montreal, QC, Canada

## Abstract

**Background:**

Patients with IBD are at risk for metabolic dysfunction-associated steatotic liver disease (MASLD) due to chronic inflammation, hepatotoxic drugs, alteration of gut microbiota. MASLD, formerly known as non-alcoholic fatty liver disease, provides a positive rather than negative diagnosis, appropriately assigns a metabolic basis for hepatic steatosis (HS), avoids any potentially stigmatizing term, and excludes alcohol abuse. MASLD carries higher risk of both liver fibrosis progression and extra-hepatic involvement, including cardiovascular disease , extra-hepatic cancer, hypothyroidism, chronic kidney disease (CKD). Data on the effect of MASLD on fibrosis progression and multi-organ co-morbidities are lacking in this population.

**Aims:**

We aimed to determine if MASLD and liver fibrosis carry a higher risk of extra-hepatic co-morbidities in IBD.

**Methods:**

We prospectively included consecutive IBD patients who underwent liver stiffness measurement (LSM) with controlled attenuation parameter (CAP) by Fibroscan at a single centre. MASLD was defined as any grade HS without alcohol abuse and viral hepatitis. HS progression was defined as any grade HS (CAPampersand:003E270 dB/m), or transition to severe HS (CAPampersand:003E330 dB/m) with CAPampersand:003E270 but ampersand:003C330 dB/m at baseline. Fibrosis progression was defined as significant liver fibrosis (LSM≥8 kPa), or transition to cirrhosis (LSM≥13 kPa) with LSMampersand:003E8 but ampersand:003C13 kPa at baseline. We estimated incidence rates of HS and fibrosis progression by dividing participants with the outcome by number of person-years (PY) of follow-up. Covariate adjustments for HS progression were evaluated by multivariable Cox regression models and predictors of extra-hepatic conditions by multivariable logistic regression analysis.

**Results:**

430 patients were included with mean age 43 years, BMI 25 Kg/m^2^, IBD duration 14 years, CRP 5.2, ALT 22; females 45%, ulcerative colitis (UC) 31.8%, T2DM 4.7%. Patients with MASLD had higher proportion of CV events (12% vs. 6%), CKD (8% vs. 3%) and hypothyroidism (12% vs. 6%) vs. those without. After adjusting for age, male sex and Crohn’s IBD subtype, MASLD remained an independent predictor of extra-hepatic comorbidities (aOR 1.79, 95% CI 1.15–2.78; p=0.01) with T2DM (aOR 3.53, 95% CI 1.68-7.42; p=0.001). Patients were followed for 26 months (SD 16.4). Rate of HS progression was 16.2 per 100 PY (95% CI, 11.5-22.8) and liver fibrosis progression was 6.12 per 100 PY (95% CI 3.48-10.44). In multivariable analysis, after adjusting for IBD duration and BMI,UC was associated with faster progression of HS (aHR 2.21, 95% CI 1.02-4.91).

**Conclusions:**

MASLD is associated with extra-hepatic diseases in patients with IBD and can progress to liver fibrosis and cirrhosis.

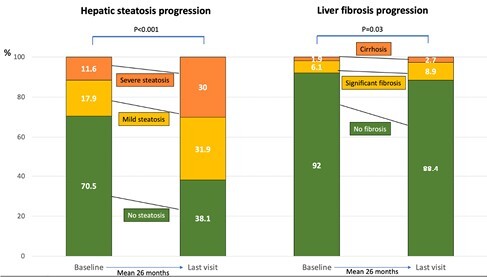

**Figure 1**. Evolution of NAFLD and associated liver fibrosis in patients with IBD.

**Funding Agencies:**

CIHR

